# Convergence Results on Iteration Algorithms to Linear Systems

**DOI:** 10.1155/2014/273873

**Published:** 2014-05-13

**Authors:** Zhuande Wang, Chuansheng Yang, Yubo Yuan

**Affiliations:** ^1^School of Mathematical Science, University of Electronic Science and Technology of China, Chengdu, Sichuan 611731, China; ^2^Department of Mathematics, Zhejiang Ocean University, Zhoushan, Zhejiang 316000, China; ^3^School of Information Science and Engineering, East China University of Science and Technology, Shanghai 200237, China

## Abstract

In order to solve the large scale linear systems, backward and Jacobi iteration algorithms are employed. The convergence is the most important issue. In this paper, a unified backward iterative matrix is proposed. It shows that some well-known iterative algorithms can be deduced with it. The most important result is that the convergence results have been proved. Firstly, the spectral radius of the Jacobi iterative matrix is positive and the one of backward iterative matrix is strongly positive (lager than a positive constant). Secondly, the mentioned two iterations have the same convergence results (convergence or divergence simultaneously). Finally, some numerical experiments show that the proposed algorithms are correct and have the merit of backward methods.

## 1. Introduction

The primal goal of this paper is to study the iterative methods of the linear systems:

(1) $$\begin{array}{c} Ax=b, \end{array}$$

where *A* is a given *n* × *n* complex or real matrix.

It is well known that linear systems arise in studies in many areas such as engineering and industrial science. For example, in the field of numerical solutions of differential-algebraic equations (DAE) and ordinary differential equations (ODE) [1–3] it is very important to solve (1). In digital image and signal processing, especially in compressed sensing, Stojnic [4] has mentioned that the systems (1) are the mathematical background of compressed sensing problems and studied sharp lower bounds on the values of allowable sparsity for any given number (proportional to the length of the unknown vector) of equations for the case of the so-called block-sparse unknown vectors. In the blind source separation of signal, Congedo et al. [5] have showed that it is very important to solve (1) and proposed a special method with joint singular value decomposition. In the field of biomedical engineering, Deo et al. [6] have mentioned that the cardiac electrical activity can be described by the bidomain equations and pointed out that the numerical solution of partial differential equations (PDEs) associated with bidomain problems often leads to (1). Moreover, they have proposed a novel preconditioner for the PCG method to solve (1) and a cheap iterative method such as successive overrelaxation (SOR) to further refine the solution for a desired accuracy. In 2008, Shou et al. [7] have showed that the reconstruction of epicardial potentials (EPs) from body surface potentials (BSPs) can be characterized as an ill-posed inverse problem and geometric errors in the ECG inverse problem will directly affect the calculation of transfer matrix *A* in (1). In the field of systems and control science, Ding and Chen [8] have pointed out that Sylvester equations in systems and control especially Lyapunov equations in continuous- and discrete-time stability analysis can be converted into equivalent equations as (1). In the field of machine learning many problems of classification and regression, such as single-hidden layer neural networks [9, 10], support vector machines, functional neural networks, and so on, can be summarized as (1). Therefore, the solution of (1) is very important in scientific computing.

The methods to solve linear systems can be roughly divided into two categories: direct methods and iterative methods 1. Iterative methods are more suitable than direct methods for large linear systems [11, 12]. The current research on iterative algorithms has been more mature, but how to make it fit the new architecture model is complicated. In order to gain more good performance, acceleration has been applied and architecture has been considered [13].

In this paper, we do some research with the iterative algorithm. To this end, the paper is organized as follows. In Section 2, we introduce the backward MPSD (backward modified preconditioned simultaneous displacement) iterative method which is a unified form of some important backward iterations. In Section 3, we first introduce some important lemmas which will be used and then we obtained the convergence results between backward MPSD iteration and Jacobi iteration. We also proposed convergence results between some backward iterations and Jacobi iteration in the corollaries. In Section 4, some examples and numerical experiments have been done to make sure of the correctness of results. Especially, we point out that the backward iteration is better than the original one in many cases just like Example 4.

## 2. A Unified Framework of Iteration Matrix and Algorithm

The basic idea to solve (1) is matrix splitting. If we let

(2) $$\begin{array}{c} A=D-C_{L}-C_{U},\;L=D^{-1}C_{L},\;\;U=D^{-1}C_{U}, \end{array}$$

where *D* = diag⁡(*A*) is a diagonal matrix obtained from *A* and nonsingular and *C*_*L*_ and *C*_*U*_ are strictly lower and upper triangular matrices obtained from *A*, (1) becomes the equivalent one:

(3) $$\begin{array}{c} \left(I-L-U\right)x=D^{-1}b. \end{array}$$

At this moment, *D* ^−1^ *A* = (*I* − *L* − *U*).

The Jacobi iterative matrix is

(4) $$\begin{array}{c} B=L+U=I-D^{-1}A. \end{array}$$

The MPSD (modified preconditioned simultaneous displacement) iterative method is studied in [14–17].

If *τ* > 0 is a real constant, obviously (3) is equivalent to

(5) $$\begin{array}{c} \tau \left(I-L-U\right)x=\tau D^{-1}b. \end{array}$$

At the same time, if *ω*_1_, *ω*_2_ are real constants, we can obtain the following equivalent from (5):

(6) (I−ω1U−ω2L+ω1ω2UL)x  =(I−ω1U−ω2L+ω1ω2UL)x   +(−τI+τL+τU)x+τD−1b.

It is easy to verify that

(7) (I−ω1U)(I−ω2L)x  =[(1−τ)I+(τ−ω1)U+(τ−ω2)L+ω1ω2UL]x   +τD−1b.

With (7), we can construct the backward MPSD iterative method as follows:

(8) $$\begin{array}{c} x^{(k+1)\;}={\tilde{S}}_{\tau ,\omega_{1},\omega_{2}}x^{\left(k\right)}+{\left(I-\omega_{2}L\right)}^{-1}{\left(I-\omega_{1}U\right)}^{-1}\tau D^{-1}b, \end{array}$$

where

(9) S~τ,ω1,ω2=(I−ω2L)−1(I−ω1U)−1×[(1−τ)I+(τ−ω1)U  +(τ−ω2)L+ω1ω2UL],

which we named as backward MPSD iterative matrix.

Also, we have the following algorithm.

*Backward MPSD Algorithm*

*Step  0 (Input).* Matrix *A*, vector *b*, *τ* > 0, *ω*_1_, *ω*_2_, algorithm stop cutoff *ϵ*. 

*Step  1 (Initialization).* Compute *D* = diag⁡(*A*), *C*_*L*_, *C*_*U*_, *L* = *D* ^−1^ *C*_*L*_, *U* = *D* ^−1^ *C*_*U*_, *x* ^(0)^ = 0, and set *i*≔0. 

*Step  2.* Compute matrix ${\tilde{S}}_{\tau ,\omega_{1},\omega_{2}}$ according to (9). 

*Step  3.* Compute *x* ^(*i*+1)^ with (8). 

*Step  4.* If ||*x* ^(*i*+1)^−*x* ^(*i*)^||_2_^2^ ⩽ *ϵ*, then stop and accept *x* ^(*i*)^ as the solution of (1); else *i* : = *i* + 1; go to step 3.

**Remark 1**

With special values of *ω*_1_,   *ω*_2_, and *τ*, we have the following.

1. When *ω*_1_ = 0, *ω*_2_ = 0, and *τ* = 1, we obtain the Jacobi iterative method.
2. When *ω*_1_ = 0, *ω*_2_ = 0, and *τ* = *ω*, we obtain the backward JOR iterative method.
3. When *ω*_1_ = 1, *ω*_2_ = 0, and *τ* = 1, we obtain the backward G-S iterative method.
4. When *ω*_1_ = *ω*, *ω*_2_ = 0, and *τ* = *ω*, we obtain the backward SOR iterative method.
5. When *ω*_1_ = *ω*, *ω*_2_ = 0, and *τ* = *α*, we obtain the backward AOR iterative method.
6. When *ω*_1_ = *ω*, *ω*_2_ = *ω*, and *τ* = *ω*(2 − *ω*), we obtain the backward SSOR iterative method.
7. When *ω*_1_ = *ω*, *ω*_2_ = *ω*, and *τ* = *ω*, we obtain the backward EMA iterative method.
8. When *ω*_1_ = *ω*, *ω*_2_ = *ω*, and *τ* = *α*, we obtain the backward PSD iterative method.
9. When *ω*_1_ = *ω*, *ω*_2_ = *ω*, and *τ* = 1, we obtain the backward PJ iterative method.

The convergence relationship between the Gauss-Seidel iterative matrix and the Jacobi iterative matrix is studied in [12], and the generalized results are studied in [18]. Some eigenvalue relationships between other iterative matrices and Jacobi iterative matrix are studied with the p-cyclic case in [19–26]. Some backward iterations are studied in [27]. In the following we consider the convergence results between the backward MPSD iterative matrix and the Jacobi iterative matrix and obtain convergence relationships between some other backward iterative matrices and Jacobi matrix.

## 3. Convergence Results

In order to obtain the convergence results, we give some well-known results which will be used in the proof of Theorem 7 as follows.

**Definition 2 (see [13])**

The splitting *A* = *M* − *N* with *A* and *M* nonsingular is called a regular splitting if *M* ^−1^ ≥ 0 and *N* ≥ 0. It is called a weak regular splitting if *M* ^−1^ ≥ 0 and *M* ^−1^ *N* ≥ 0.

It is obvious that a regular splitting is a weak regular splitting.

**Lemma 3 (see [13])**

The nonnegative matrix *T* ∈ *R* ^*n*×*n*^ is convergent; that is, *ρ*(*T*) < 1 if and only if (*I* − *T*)^−1^ exists and (*I* − *T*)^−1^ = ∑_*k*=1_^*∞*^ *T* ^*k*^ ≥ 0.

**Lemma 4 (see [13])**

Let *A* = *M* − *N* be a weak regular splitting of *A*, *H* = *M* ^−1^ *N*. Then the following statements are equivalent.

 
*(1)* *A* ^−1^ ≥ 0; that is, *A* is inverse-positive.
 
*(2)* *A* ^−1^ *N* ≥ 0.
 
*(3)* *ρ*(*H*) = *ρ*(*A* ^−1^ *N*)/(1 + *ρ*(*A* ^−1^ *N*)) so that *ρ*(*H*) < 1.

**Lemma 5 (see [12])**

Let *A* ≥ 0 be an irreducible *n* × *n* matrix. Then

 
*(1)* *A* has a positive real eigenvalue equal to its spectral radius;
 
*(2)* to *ρ*(*A*), there corresponds an eigenvector *x* > 0,
 
*(3)* *ρ*(*A*) increases when any entry of *A* increases,
 
*(4)* *ρ*(*A*) is a simple eigenvalue of *A*.

**Lemma 6 (see [12])**

Let *A* = (*a*_*ij*_) ≥ 0 be an irreducible *n* × *n* matrix. Then for any *x* > 0, either

(10) $$\begin{array}{c} \underset{1\leq i\leq n}{\min}\frac{\sum_{j=1}^{n}a_{ij}x_{j}}{x_{i}}<\rho \left(A\right)<\underset{1\leq i\leq n}{\max}\frac{\sum_{j=1}^{n}a_{ij}x_{j}}{x_{i}} \end{array}$$

or

(11) $$\begin{array}{c} \frac{\sum_{j=1}^{n}a_{ij}x_{j}}{x_{i}}=\rho \left(A\right)\;\forall i. \end{array}$$

By the lemmas above, we give the convergence theorem in the following.

**Theorem 7**

Let the coefficient matrix *A* of (1) be irreducible with *a*_*ii*_ ≠ 0, ∀ *i*, *B* = *U* + *L* ≥ 0 the Jacobi matrix, and ${\tilde{S}}_{\tau ,\omega_{1},\omega_{2}}$ the backward MPSD iterative matrix. Then, for 0 ≤ *ω*_*k*_ < *τ* ≤ 1, *k* = 1,2, we have the following.

 
*(1)* *ρ*(*B*) > 0, $\rho ({\tilde{S}}_{\tau ,\omega_{1},\omega_{2}})>1-\tau$.
 (2) One and only one of the following mutually exclusive relations is valid.
  (i) $0<\rho (B)<1\Leftrightarrow 1-\tau <\rho ({\tilde{S}}_{\tau ,\omega_{1},\omega_{2}})<1$.
  (ii) $\rho (B)=1\Leftrightarrow \rho ({\tilde{S}}_{\tau ,\omega_{1},\omega_{2}})=1$.
  (iii) $\rho (B)>1\Leftrightarrow \rho ({\tilde{S}}_{\tau ,\omega_{1},\omega_{2}})>1$.

Thus, The Jacobi iterative method and the backward MPSD iterative method are either both convergent or both divergent.

**Proof**

Combining *ρ*(*ω*_2_ *L*) = *ρ*(*ω*_1_ *U*) = 0 with Lemma 3, we have (*I* − *ω*_2_ *L*)^−1^ ≥ 0, (*I* − *ω*_1_ *U*)^−1^ ≥ 0, and

(12) S~τ,ω1,ω2=(I−ω2L)−1(I−ω1U)−1  ×[(1−τ)I+(τ−ω1)U    +(τ−ω2)L+ω1ω2UL]=(I+ω2L+ω22L2+⋯)  ×(I+ω1U+ω12U2+⋯)  ×[(1−τ)I+(τ−ω1)U    +(τ−ω2)L+ω1ω2UL]≥(1−τ)I+(τ−ω1)U+(τ−ω2)L.

Since *a*_*ii*_ ≠ 0 and *A* is irreducible, *I* − *L* − *U* = *D* ^−1^ *A* and *B* = *L* + *U* are irreducible. By 0 ≤ *ω*_*k*_ < *τ* ≤ 1, *k* = 1,2, we have (1 − *τ*)*I* + (*τ* − *ω*_1_)*U* + (*τ* − *ω*_2_)*L* ≥ 0 and irreducible. Thus, by (12), ${\tilde{S}}_{\tau ,\omega_{1},\omega_{2}}\geq 0$ and is irreducible. By Lemma 5, there exists $\lambda =\rho ({\tilde{S}}_{\tau ,\omega_{1},\omega_{2}})>0$ and corresponding vector *x* = (*x*_1_, *x*_2_,…, *x*_*n*_)^*T*^ > 0, such that ${\tilde{S}}_{\tau ,\omega_{1},\omega_{2}}x=\lambda x$; namely,

(13) [(1−τ)I+(τ−ω1)U+(τ−ω2)L+ω1ω2UL]x  =λ(I−ω1U)(I−ω2L)x.

Let *η*_*k*_ = *τ* − *ω*_*k*_ + *λω*_*k*_, *k* = 1,2; by calculation,

(14) $$\begin{array}{c} \eta_{1}Ux+\eta_{2}Lx+\left(1-\lambda \right)\omega_{1}\omega_{2}ULx=\left[\lambda -\left(1-\tau \right)\right]x; \end{array}$$

that is,

(15) $$\begin{array}{c} \eta_{1}Ux+\eta_{2}Lx=\left(\lambda -1\right)\omega_{1}\omega_{2}ULx+\left[\lambda -\left(1-\tau \right)\right]x. \end{array}$$

(1) Since *B* ≥ 0 is irreducible, by Lemma 5, *ρ*(*B*) > 0. If $\rho ({\tilde{S}}_{\tau ,\omega_{1},\omega_{2}})\geq 1$, by 0 ≤ *ω*_*k*_ < *τ* ≤ 1, *k* = 1,2, $\rho ({\tilde{S}}_{\tau ,\omega_{1},\omega_{2}})\geq 1>1-\tau$. If $\rho ({\tilde{S}}_{\tau ,\omega_{1},\omega_{2}})<1$, then *λ* − (1 − *τ*) ≥ 0 because the left side of (14) is nonnegative, Thus *λ* ≥ 1 − *τ*. By (14),

(16) $$\begin{array}{c} \eta_{1}Ux+\eta_{2}Lx\leq \left[\lambda -\left(1-\tau \right)\right]x. \end{array}$$

If *λ* = 1 − *τ*, by (16), we have *η*_1_ *Ux* + *η*_2_ *Lx* ≤ 0; that is,

(17) $$\begin{array}{c} \eta_{1}\overset{n}{\underset{j=i+1}{\sum }}b_{ij}x_{j}+\eta_{2}\overset{i-1}{\underset{j=1}{\sum }}b_{ij}x_{j}\leq 0,\;\forall i. \end{array}$$

Since *η*_*k*_ > 0, *k* = 1,2, *B* = (*b*_*ij*_) ≥ 0 and *x* > 0, we obtain that *B* = 0. Thus, *ρ*(*B*) = 0. This contradicts *ρ*(*B*) > 0. So, $\rho ({\tilde{S}}_{\tau ,\omega_{1},\omega_{2}})>1-\tau$.

(2) For mutually exclusive relations, consider the following.

(i) If 0 < *ρ*(*B*) < 1, let

(18) M=(I−ω1U)(I−ω2L),N=[(1−τ)I+(τ−ω1)U+(τ−ω2)L+ω1ω2UL],

and then

(19) $$\begin{array}{c} {\tilde{S}}_{\tau ,\omega_{1},\omega_{2}}=M^{-1}N. \end{array}$$

Since *M* ^−1^ = (*I* − *ω*_2_ *L*)^−1^(*I* − *ω*_1_ *U*)^−1^ ≥ 0 and *N* ≥ 0, *T* = *M* − *N* is a regular splitting:

(20) $$\begin{array}{c} T=M-N=\tau I-\tau \left(U+L\right)=\tau \left(I-\left(U+L\right)\right)=\tau \left(I-B\right). \end{array}$$

By *B* ≥ 0, 0 < *ρ*(*B*) < 1, and 0 < *τ* ≤ 1, we know that *T* ^−1^ = (1/*τ*)(*I* − *B*)^−1^ ≥ 0. By Lemma 4, $\rho ({\tilde{S}}_{\tau ,\omega_{1},\omega_{2}})=\rho (M^{-1}N)<1$. Combine this with the result in (1), we have $1-\tau <\rho ({\tilde{S}}_{\tau ,\omega_{1},\omega_{2}})<1$.

If $1-\tau <\lambda =\rho ({\tilde{S}}_{\tau ,\omega_{1},\omega_{2}})<1$, by (14), we have

(21) $$\begin{array}{c} \eta_{1}Ux+\eta_{2}Lx\leq \left[\lambda -\left(1-\tau \right)\right]x. \end{array}$$

Since *η*_*k*_ > 0, *k* = 1,2, *b*_*ii*_ = 0, ∀ *i*,

(22) min⁡k=1,2 ηk(∑j=1nbijxj)≤η1∑j=i+1nbijxj+η2∑j=1i−1bijxj≤[λ−(1−τ)]xi, ∀i;

that is,

(23) $$\begin{array}{c} \frac{\sum_{j=1}^{n}b_{ij}x_{j}}{x_{i}}\leq \frac{\lambda -\left(1-\tau \right)}{\min_{k=1,2}\;\eta_{k}},\;\forall i. \end{array}$$

By *λ* < 1 and 1 − *ω*_*k*_ > 0  (*k* = 1,2), there is

(24) $$\begin{array}{c} \lambda \left(1-\omega_{k}\right)<1-\omega_{k},\;\;\lambda -1<-\omega_{k}+\lambda \omega_{k},\\ 0<\lambda -\left(1-\tau \right)=\lambda -1+\tau <\eta_{k}. \end{array}$$

Thus,

(25) $$\begin{array}{c} 0<\frac{\lambda -\left(1-\tau \right)}{\eta_{k}}<1,\;k=1,2. \end{array}$$

Combining (23) with (29), we have

(26) $$\begin{array}{c} \frac{\sum_{j=1}^{n}b_{ij}x_{j}}{x_{i}}<1,\;\forall i. \end{array}$$

By Lemma 6, we obtain that 0 < *ρ*(*B*) < 1.

(ii) If $\lambda =\rho ({\tilde{S}}_{\tau ,\omega_{1},\omega_{2}})=1$, by (14), we have

(27) $$\begin{array}{c} \tau \left(U+L\right)x=\left[1-\left(1-\tau \right)\right]x=\tau x; \end{array}$$

namely, *Bx* = *x*. Since *x* > 0, we have

(28) $$\begin{array}{c} \frac{\sum_{j=1}^{n}b_{ij}x_{j}}{x_{i}}=1,\;\forall i. \end{array}$$

By Lemma 6, we obtain that *ρ*(*B*) = 1.

(iii) If $\lambda =\rho ({\tilde{S}}_{\tau ,\omega_{1},\omega_{2}})>1$, by (15), we have

(29) $$\begin{array}{c} \eta_{1}Ux+\eta_{2}Lx\geq \left[\lambda -\left(1-\tau \right)\right]x. \end{array}$$

Since *η*_*k*_ > 0, *k* = 1,2, *b*_*ii*_ = 0, ∀ *i*,

(30) max⁡k=1,2 ηk(∑j=1nbijxj)≥η1∑j=i+1nbijxj+η2∑j=1i−1bijxj≥[λ−(1−τ)]xi, ∀i;

that is,

(31) $$\begin{array}{c} \frac{\sum_{j=1}^{n}b_{ij}x_{j}}{x_{i}}\geq \frac{\lambda -\left(1-\tau \right)}{\max_{k=1,2}\;\eta_{k}},\;\forall i. \end{array}$$

By *λ* > 1 and 1 − *ω*_*k*_ > 0  (*k* = 1,2), there is

(32) $$\begin{array}{c} \lambda \left(1-\omega_{k}\right)>1-\omega_{k},\;\;\lambda -1>-\omega_{k}+\lambda \omega_{k},\\ \lambda -\left(1-\tau \right)=\lambda -1+\tau >\eta_{k}>0. \end{array}$$

Thus,

(33) $$\begin{array}{c} \frac{\lambda -\left(1-\tau \right)}{\eta_{k}}>1,\;k=1,2. \end{array}$$

Combining (31) with (33), we have

(34) $$\begin{array}{c} \frac{\sum_{j=1}^{n}b_{ij}x_{j}}{x_{i}}>1,\;\forall i. \end{array}$$

By Lemma 6, we obtain that *ρ*(*B*) > 1.

If *ρ*(*B*) = 1 and $\rho ({\tilde{S}}_{\tau ,\omega_{1},\omega_{2}})\neq 1$, by (1), we obtain that $1-\tau <\lambda =\rho ({\tilde{S}}_{\tau ,\omega_{1},\omega_{2}})<1$ or $\rho ({\tilde{S}}_{\tau ,\omega_{1},\omega_{2}})>1$. Thus, by (i) and (iii), we know that 0 < *ρ*(*B*) < 1 or *ρ*(*B*) > 1. This contradicts *ρ*(*B*) = 1. So, $\rho ({\tilde{S}}_{\tau ,\omega_{1},\omega_{2}})=1$.

If *ρ*(*B*) > 1 and $\rho ({\tilde{S}}_{\tau ,\omega_{1},\omega_{2}})\leq 1$, by (i) and (ii), we have *ρ*(*B*) ≤ 1. This contradicts *ρ*(*B*) > 1. So, $\rho ({\tilde{S}}_{\tau ,\omega_{1},\omega_{2}})>1$.

With special values of *ω*_1_, *ω*_2_, and *τ*, we have the following corollaries.

**Corollary 8**

Let the coefficient matrix *A* of (1) be irreducible, *B* = *U* + *L* ≥ 0 the Jacobi matrix, and ${\tilde{S}}_{\omega ,0,0}$ the backward JOR iterative matrix. Then, for 0 ≤ *ω* ≤ 1, we have the following.

 
*(1)* *ρ*(*B*) > 0, $\rho ({\tilde{S}}_{\omega ,0,0})>1-\omega$.
 
*(2)* One and only one of the following mutually exclusive relations is valid. 
  (i) $0<\rho (B)<1\Leftrightarrow 1-\omega <\rho ({\tilde{S}}_{\omega ,0,0})<1$.
  (ii) $\rho (B)=1\Leftrightarrow \rho ({\tilde{S}}_{\omega ,0,0})=1$.
  (iii) $\rho (B)>1\Leftrightarrow \rho ({\tilde{S}}_{\omega ,0,0})>1$.

Thus, The Jacobi iterative method and the backward JOR iterative method are either both convergent or both divergent.

**Corollary 9**

Let the coefficient matrix *A* of (1) be irreducible, *B* = *U* + *L* ≥ 0 the Jacobi matrix, and ${\tilde{S}}_{1,1,0}$ the backward Gauss-Seidel iterative matrix. Then, we have the following.

 
*(1)* *ρ*(*B*) > 0, $\rho ({\tilde{S}}_{1,1,0})>0$.
 
*(2)* One and only one of the following mutually exclusive relations is valid.
  - $0<\rho (B)<1\Leftrightarrow 0<\rho ({\tilde{S}}_{1,1,0})<1$.
  - $\rho (B)=1\Leftrightarrow \rho ({\tilde{S}}_{1,1,0})=1$.
  - $\rho (B)>1\Leftrightarrow \rho ({\tilde{S}}_{1,1,0})>1$.

Thus, The Jacobi iterative method and the backward Gauss-Seidel iterative method are either both convergent or both divergent.

**Corollary 10**

Let the coefficient matrix *A* of (1) be irreducible, *B* = *U* + *L* ≥ 0 the Jacobi matrix, and ${\tilde{S}}_{\omega ,\omega ,0}$ the backward SOR iterative matrix. Then, for 0 ≤ *ω* ≤ 1, we have the following.

 
*(1)* *ρ*(*B*) > 0, $\rho ({\tilde{S}}_{\omega ,,\omega ,0})>1-\omega$.
 
*(2)* One and only one of the following mutually exclusive relations is valid.  
  - $0<\rho (B)<1\Leftrightarrow 1-\omega <\rho ({\tilde{S}}_{\omega ,,\omega ,0})<1$.
  - $\rho (B)=1\Leftrightarrow \rho ({\tilde{S}}_{\omega ,\omega ,0})=1$.
  - $\rho (B)>1\Leftrightarrow \rho ({\tilde{S}}_{\omega ,\omega ,0})>1$.

Thus, The Jacobi iterative method and the backward SOR iterative method are either both convergent or both divergent.

**Corollary 11**

Let the coefficient matrix *A* of (1) be irreducible, *B* = *U* + *L* ≥ 0 the Jacobi matrix, and ${\tilde{S}}_{\alpha ,\omega ,0}$ the backward AOR iterative matrix. Then, for 0 ≤ *ω* < *α* ≤ 1, we have the following.

 
*(1)* *ρ*(*B*) > 0, $\rho ({\tilde{S}}_{\alpha ,\omega ,0})>1-\alpha$.
 
*(2)* One and only one of the following mutually exclusive relations is valid.
  (i) $0<\rho (B)<1\Leftrightarrow 1-\alpha <\rho ({\tilde{S}}_{\alpha ,\omega ,0})<1$.
  (ii) $\rho (B)=1\Leftrightarrow \rho ({\tilde{S}}_{\alpha ,\omega ,0})=1$.
  (iii) $\rho (B)>1\Leftrightarrow \rho ({\tilde{S}}_{\alpha ,\omega ,0})>1$.

Thus, The Jacobi iterative method and the backward AOR iterative method are either both convergent or both divergent.

**Corollary 12**

Let the coefficient matrix *A* of (1) be irreducible, *B* = *U* + *L* ≥ 0 the Jacobi matrix, and ${\tilde{S}}_{\omega (2-\omega ),\omega ,\omega }$ the backward SSOR iterative matrix. Then, for 0 ≤ *ω* ≤ 1, we have the following.

 
*(1)* *ρ*(*B*) > 0, $\rho ({\tilde{S}}_{\omega (2-\omega ),\omega ,\omega })>{\left(1-\omega \right)}^{2}$.
 
*(2)* One and only one of the following mutually exclusive relations is valid.  
  - $0<\rho (B)<1\Leftrightarrow {\left(1-\omega \right)}^{2}<\rho ({\tilde{S}}_{\omega (2-\omega ),\omega ,\omega })<1$.
  - $\rho (B)=1\Leftrightarrow \rho ({\tilde{S}}_{\omega (2-\omega ),\omega ,\omega })=1$.
  - $\rho (B)>1\Leftrightarrow \rho ({\tilde{S}}_{\omega (2-\omega ),\omega ,\omega })>1$.

Thus, The Jacobi iterative method and the backward SSOR iterative method are either both convergent or both divergent.

**Corollary 13**

Let the coefficient matrix *A* of (1) be irreducible, *B* = *U* + *L* ≥ 0 the Jacobi matrix, and ${\tilde{S}}_{\omega ,\omega ,\omega }$ the backward EMA iterative matrix. Then, for 0 ≤ *ω* ≤ 1, we have the following.

 
*(1)* *ρ*(*B*) > 0, $\rho ({\tilde{S}}_{\omega ,\omega ,\omega })>{\left(1-\omega \right)}^{2}$.
 
*(2)* One and only one of the following mutually exclusive relations is valid.  
  (i) $0<\rho (B)<1\Leftrightarrow {\left(1-\omega \right)}^{2}<\rho ({\tilde{S}}_{\omega ,\omega ,\omega })<1$.
  (ii) $\rho (B)=1\Leftrightarrow \rho ({\tilde{S}}_{\omega ,\omega ,\omega })=1$.
  (iii) $\rho (B)>1\Leftrightarrow \rho ({\tilde{S}}_{\omega ,\omega ,\omega })>1$.

Thus, The Jacobi iterative method and the backward EMA iterative method are either both convergent or both divergent.

**Corollary 14**

Let the coefficient matrix *A* of (1) be irreducible, *B* = *U* + *L* ≥ 0 the Jacobi matrix, and ${\tilde{S}}_{\alpha ,\omega ,\omega }$ the backward PSD iterative matrix. Then, for 0 ≤ *ω* < *α* ≤ 1, we have the following.

 
*(1)* *ρ*(*B*) > 0, $\rho ({\tilde{S}}_{\alpha ,\omega ,\omega })>1-\alpha$.
 
*(2)* One and only one of the following mutually exclusive relations is valid.
  (i) $0<\rho (B)<1\Leftrightarrow 1-\alpha <\rho ({\tilde{S}}_{\alpha ,\omega ,0})<1$.
  (ii) $\rho (B)=1\Leftrightarrow \rho ({\tilde{S}}_{\alpha ,\omega ,\omega })=1$.
  (iii) $\rho (B)>1\Leftrightarrow \rho ({\tilde{S}}_{\alpha ,\omega ,\omega })>1$.

Thus, The Jacobi iterative method and the backward PSD iterative method are either both convergent or both divergent.

**Corollary 15**

Let the coefficient matrix *A* of (1) be irreducible, *B* = *U* + *L* ≥ 0 the Jacobi matrix, and ${\tilde{S}}_{1,\omega ,\omega }$ the backward PJ iterative matrix. Then, for 0 ≤ *ω* ≤ 1, we have the following.

 
*(1)* *ρ*(*B*) > 0, $\rho ({\tilde{S}}_{1,\omega ,\omega })>0$.
 
*(2)* One and only one of the following mutually exclusive relations is valid.    
  (i) $0<\rho (B)<1\Leftrightarrow 0<\rho ({\tilde{S}}_{1,\omega ,\omega })<1$.
  (ii) $\rho (B)=1\Leftrightarrow \rho ({\tilde{S}}_{1,\omega ,\omega })=1$.
  (iii) $\rho (B)>1\Leftrightarrow \rho ({\tilde{S}}_{1,\omega ,\omega })>1$.

Thus, The Jacobi iterative method and the backward PJ iterative method are either both convergent or both divergent.

**Remark 16**

The convergence results between the backward MPSD and Jacobi iterative matrix are proposed, and The convergence results between some special cases of backward MPSD (such as backward JOR, backward G-S, backward EMA, and backward PSD) and Jacobi iterative matrix are obtained. These results involve some special iterative methods which are proposed in the references.

## 4. Numerical Examples

In this section, we show five examples. The first three examples are used to show the convergence of the proposed iterative methods. Example 4 is used to show the divergence of the proposed iterative methods.  Example 5 shows that the backward iterative methods are better than the origin methods when the upper triangular part dominates the lower triangular part. In the following figures, horizontal axis denotes the numbers of iterations and vertical axis denotes the errors of iterations.

**Example 1**

Let the coefficient matrix *A* and the vector *b* of (1) be

(35) $$\begin{array}{c} A=\left[\begin{array}{cccc} 1 & 0 & -\frac{1}{4} & -\frac{1}{4}\\ 0 & 1 & -\frac{1}{4} & -\frac{1}{4}\\ -\frac{1}{4} & -\frac{1}{4} & 1 & 0\\ -\frac{1}{4} & -\frac{1}{4} & 0 & 1 \end{array}\right],\;\;b=\left[\begin{array}{c} 1\\ 1\\ 1\\ 1 \end{array}\right]. \end{array}$$

The Jacobi iterative matrix is

(36) $$\begin{array}{c} B=\left[\begin{array}{cccc} 0 & 0 & \frac{1}{4} & \frac{1}{4}\\ 0 & 0 & \frac{1}{4} & \frac{1}{4}\\ \frac{1}{4} & \frac{1}{4} & 0 & 0\\ \frac{1}{4} & \frac{1}{4} & 0 & 0 \end{array}\right]. \end{array}$$

By caculation, we obtain 0 < *ρ*(*B*) = 1/2 < 1.

(1) Let *τ* = *α* = 1/2, *ω*_1_ = *ω*_2_ = *ω* = 1/4. We obtain the backward PSD iterative matrix ${\tilde{S}}_{\alpha ,\omega ,\omega }={\tilde{S}}_{1/2,1/4,1/4}$, and
  (37) $$\begin{array}{c} 1>\rho \left({\tilde{S}}_{\alpha ,\omega ,\omega }\right)=\rho \left({\tilde{S}}_{1/2,1/4,1/4}\right)=\frac{967}{1349}>\frac{1}{2}=1-\alpha . \end{array}$$
(2) Let *τ* = 1, *ω*_1_ = *ω*_2_ = *ω* = 1/2. We obtain the backward PJ iterative matrix ${\tilde{S}}_{1,\omega ,\omega }={\tilde{S}}_{1,1/2,1/2}$, and
  (38) $$\begin{array}{c} 1>\rho \left({\tilde{S}}_{1,\omega ,\omega }\right)=\rho \left({\tilde{S}}_{1,1/2,1/2}\right)=\frac{250}{693}>0=1-\tau . \end{array}$$
(3) Let *τ* = *ω* = 1/2, *ω*_1_ = *ω*_2_ = 0. We obtain the backward JOR iterative matrix ${\tilde{S}}_{\omega ,0,0}={\tilde{S}}_{1/2,0,0}$, and
  (39) $$\begin{array}{c} 1>\rho \left({\tilde{S}}_{\omega ,0,0}\right)=\rho \left({\tilde{S}}_{1/2,0,0}\right)=\frac{3}{4}>\frac{1}{2}=1-\omega . \end{array}$$
(4) Let *τ* = *ω*_1_ = *ω*_2_ = *ω* = 1/2. We obtain the backward EMA iterative matrix ${\tilde{S}}_{\omega ,\omega ,\omega }={\tilde{S}}_{1/2,1/2,1/2}$, and
  (40) $$\begin{array}{c} 1>\rho \left({\tilde{S}}_{\omega ,\omega ,\omega }\right)=\rho \left({\tilde{S}}_{1/2,1/2,1/2}\right)=\frac{943}{1386}>\frac{1}{4}={\left(1-\omega \right)}^{2}. \end{array}$$

With these iterative methods and the presented algorithm, the solution is **x** = (2,2, 2,2)^*T*^.

**Example 2**

**Figure 1:**
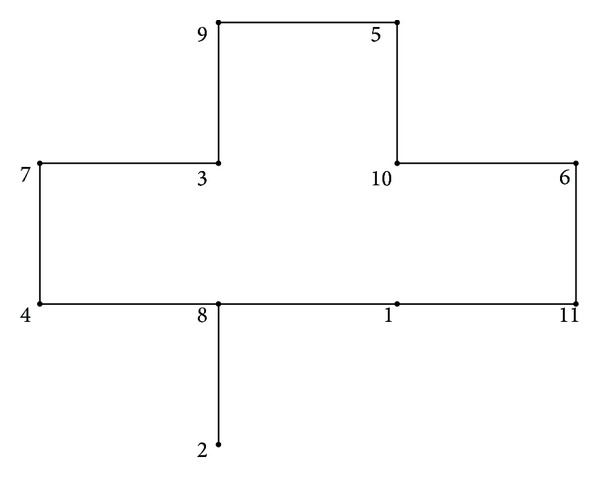
Uniform square mesh of five-point difference.

In order to obtain the numerical solution of the laplace equation

(41) $$\begin{array}{c} \frac{\partial^{2}u\left(x,y\right)}{\partial x^{2}}+\frac{\partial^{2}u\left(x,y\right)}{\partial y^{2}}=u_{xx}\left(x,y\right)+u_{yy}\left(x,y\right)=0, \end{array}$$

under a uniform square mesh of five-point difference approximations, and the interior mesh points as shown in Figure 1 [28], we can obtain the linear system (1), where the matrix *A* and the vector *b* of (1) are

(42) $$\begin{array}{c} A=\left[\begin{array}{ccccccccccc} 1 & 0 & 0 & 0 & 0 & 0 & 0 & -\frac{1}{4} & 0 & -\frac{1}{4} & -\frac{1}{4}\\ 0 & 1 & 0 & 0 & 0 & 0 & 0 & -\frac{1}{4} & 0 & 0 & 0\\ 0 & 0 & 1 & 0 & 0 & 0 & -\frac{1}{4} & -\frac{1}{4} & -\frac{1}{4} & -\frac{1}{4} & 0\\ 0 & 0 & 0 & 1 & 0 & 0 & -\frac{1}{4} & -\frac{1}{4} & 0 & 0 & 0\\ 0 & 0 & 0 & 0 & 1 & 0 & 0 & 0 & -\frac{1}{4} & -\frac{1}{4} & 0\\ 0 & 0 & 0 & 0 & 0 & 1 & 0 & 0 & 0 & -\frac{1}{4} & -\frac{1}{4}\\ 0 & 0 & -\frac{1}{4} & -\frac{1}{4} & 0 & 0 & 1 & 0 & 0 & 0 & 0\\ -\frac{1}{4} & -\frac{1}{4} & -\frac{1}{4} & -\frac{1}{4} & 0 & 0 & 0 & 1 & 0 & 0 & 0\\ 0 & 0 & -\frac{1}{4} & 0 & -\frac{1}{4} & 0 & 0 & 0 & 1 & 0 & 0\\ -\frac{1}{4} & 0 & -\frac{1}{4} & 0 & -\frac{1}{4} & -\frac{1}{4} & 0 & 0 & 0 & 1 & 0\\ -\frac{1}{4} & 0 & 0 & 0 & 0 & -\frac{1}{4} & 0 & 0 & 0 & 0 & 1 \end{array}\right],\;\;b=\left[\begin{array}{c} 1\\ 1\\ 1\\ 1\\ 1\\ 1\\ 1\\ 1\\ 1\\ 1\\ 1 \end{array}\right]. \end{array}$$

The Jacobi iterative matrix is

(43) $$\begin{array}{c} B=\left[\begin{array}{ccccccccccc} 0 & 0 & 0 & 0 & 0 & 0 & 0 & \frac{1}{4} & 0 & \frac{1}{4} & \frac{1}{4}\\ 0 & 0 & 0 & 0 & 0 & 0 & 0 & \frac{1}{4} & 0 & 0 & 0\\ 0 & 0 & 0 & 0 & 0 & 0 & \frac{1}{4} & \frac{1}{4} & \frac{1}{4} & \frac{1}{4} & 0\\ 0 & 0 & 0 & 0 & 0 & 0 & \frac{1}{4} & \frac{1}{4} & 0 & 0 & 0\\ 0 & 0 & 0 & 0 & 0 & 0 & 0 & 0 & \frac{1}{4} & \frac{1}{4} & 0\\ 0 & 0 & 0 & 0 & 0 & 0 & 0 & 0 & 0 & \frac{1}{4} & -\frac{1}{4}\\ 0 & 0 & \frac{1}{4} & \frac{1}{4} & 0 & 0 & 1 & 0 & 0 & 0 & 0\\ \frac{1}{4} & \frac{1}{4} & \frac{1}{4} & \frac{1}{4} & 0 & 0 & 0 & 0 & 0 & 0 & 0\\ 0 & 0 & \frac{1}{4} & 0 & \frac{1}{4} & 0 & 0 & 0 & 1 & 0 & 0\\ \frac{1}{4} & 0 & \frac{1}{4} & 0 & \frac{1}{4} & \frac{1}{4} & 0 & 0 & 0 & 0 & 0\\ \frac{1}{4} & 0 & 0 & 0 & 0 & \frac{1}{4} & 0 & 0 & 0 & 0 & 0 \end{array}\right]. \end{array}$$

By caculation, we obtain 0 < *ρ*(*B*) = 1159/1601 < 1.

(1) Let *τ* = *α* = 1/2, *ω*_1_ = *ω*_2_ = *ω* = 1/4. We obtain the backward PSD iterative matrix ${\tilde{S}}_{\alpha ,\omega ,\omega }={\tilde{S}}_{1/2,1/4,1/4}$, and
  (44) $$\begin{array}{c} 1>\rho \left({\tilde{S}}_{\alpha ,\omega ,\omega }\right)=\rho \left({\tilde{S}}_{1/2,1/4,1/4}\right)=\frac{1061}{1271}>\frac{1}{2}=1-\alpha . \end{array}$$
(2) Let *τ* = 1, *ω*_1_ = *ω*_2_ = *ω* = 1/2. We obtain the backward PJ iterative matrix ${\tilde{S}}_{1,\omega ,\omega }={\tilde{S}}_{1,1/2,1/2}$, and
  (45) $$\begin{array}{c} 1>\rho \left({\tilde{S}}_{1,\omega ,\omega }\right)=\rho \left({\tilde{S}}_{1,1/2,1/2}\right)=\frac{1319}{2168}>0=1-\tau . \end{array}$$
(3) Let *τ* = *ω* = 1/2, *ω*_1_ = *ω*_2_ = 0. We obtain the backward JOR iterative matrix ${\tilde{S}}_{\omega ,0,0}={\tilde{S}}_{1/2,0,0}$, and
  (46) $$\begin{array}{c} 1>\rho \left({\tilde{S}}_{\omega ,0,0}\right)=\rho \left({\tilde{S}}_{1/2,0,0}\right)=\frac{1380}{1601}>\frac{1}{2}=1-\omega . \end{array}$$
(4) Let *τ* = *ω*_1_ = *ω*_2_ = *ω* = 1/2. We obtain the backward EMA iterative matrix ${\tilde{S}}_{\omega ,\omega ,\omega }={\tilde{S}}_{1/2,1/2,1/2}$, and
  (47) $$\begin{array}{c} 1>\rho \left({\tilde{S}}_{\omega ,\omega ,\omega }\right)=\rho \left({\tilde{S}}_{1/2,1/2,1/2}\right)=\frac{3602}{4479}>\frac{1}{4}={\left(1-\omega \right)}^{2}. \end{array}$$

**Figure 2:**
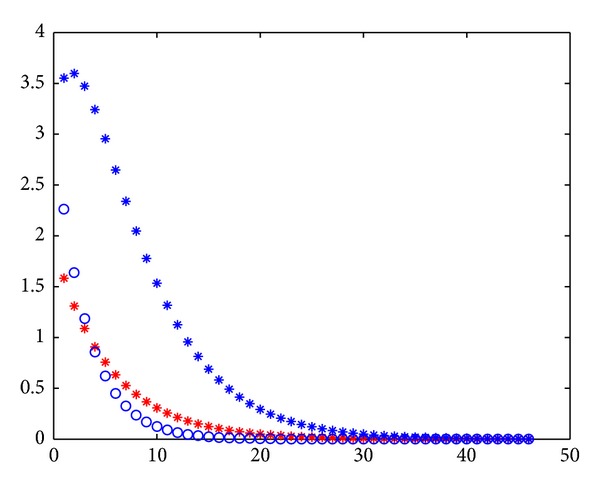
The errors of PSD and Jacobi iteration.

**Figure 3:**
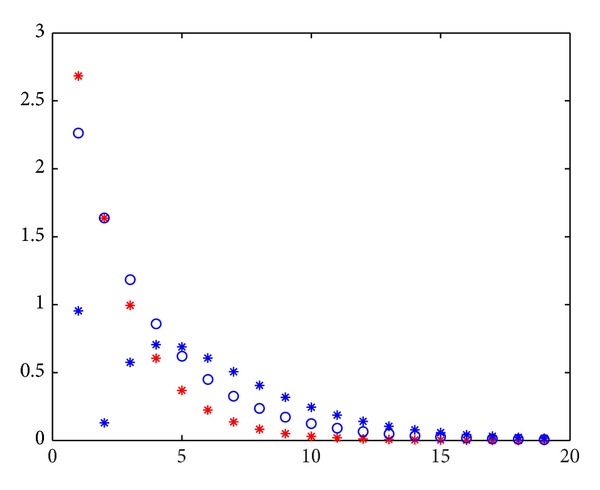
The errors of PJ and Jacobi iteration.

**Figure 4:**
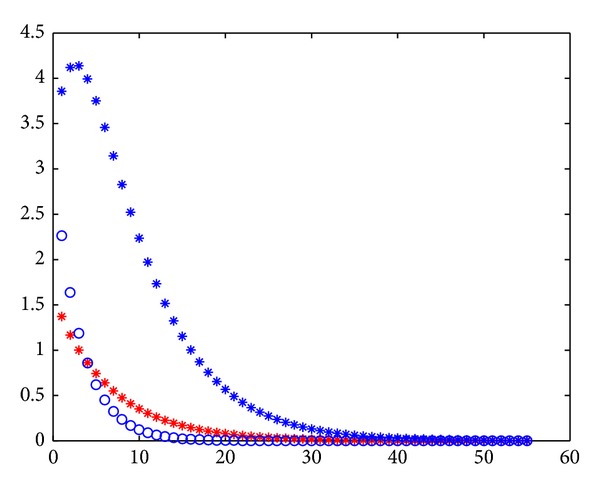
The errors of JOR and Jacobi iteration.

**Figure 5:**
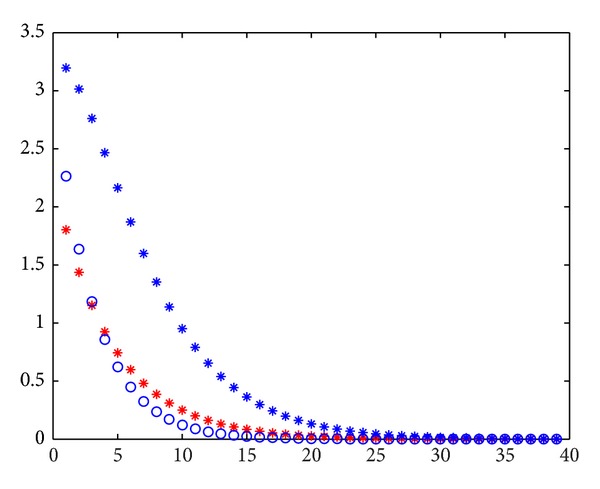
The errors of EMA and Jacobi iteration.

From Figures 2, 3, 4, and 5, the errors of Jacobi iteration are denoted by blue circles and that of MPSD iteration is denoted by red stars. By the figures above, We know that Jacobi iteration is better than backward PSD, JOR, and EMA iteration and is worse than PJ iteration under the values of *ω*_1_, *ω*_2_, and *τ* in this example.

**Example 3**

Let the coefficient matrix *A* of (1) be

(48) $$\begin{array}{c} A=\left[\begin{array}{cccccc} 1 & q & r & s & q & \cdots \\ s & 1 & q & r & \ddots & q\\ q & s & \ddots & \ddots & \ddots & s\\ r & \ddots & \ddots & 1 & q & r\\ s & \ddots & q & s & 1 & q\\ \cdots & s & r & q & s & 1 \end{array}\right], \end{array}$$

where *q* = −*p*/*n*, *r* = −*p*/(*n* + 1), and *s* = −*p*/(*n* + 2) [28]. Here, we let *n* = 100 and *p* = 1. By caculation, we obtain 0 < *ρ*(*B*) = 199/203 < 1.

(1) Let *τ* = *α* = 1/2, *ω*_1_ = *ω*_2_ = *ω* = 1/4. We obtain the backward PSD iterative matrix ${\tilde{S}}_{\alpha ,\omega ,\omega }={\tilde{S}}_{1/2,1/4,1/4}$, and
  (49) $$\begin{array}{c} 1>\rho \left({\tilde{S}}_{\alpha ,\omega ,\omega }\right)=\rho \left({\tilde{S}}_{1/2,1/4,1/4}\right)=\frac{233}{236}>\frac{1}{2}=1-\alpha . \end{array}$$
(2) Let *τ* = 1, *ω*_1_ = *ω*_2_ = *ω* = 1/2. We obtain the backward PJ iterative matrix ${\tilde{S}}_{1,\omega ,\omega }={\tilde{S}}_{1,1/2,1/2}$, and
  (50) $$\begin{array}{c} 1>\rho \left({\tilde{S}}_{1,\omega ,\omega }\right)=\rho \left({\tilde{S}}_{1,1/2,1/2}\right)=\frac{724}{749}>0=1-\tau . \end{array}$$
(3) Let *τ* = *ω* = 1/2, *ω*_1_ = *ω*_2_ = 0. We obtain the backward JOR iterative matrix ${\tilde{S}}_{\omega ,0,0}={\tilde{S}}_{1/2,0,0}$, and
  (51) $$\begin{array}{c} 1>\rho \left({\tilde{S}}_{\omega ,0,0}\right)=\rho \left({\tilde{S}}_{1/2,0,0}\right)=\frac{199}{203}>\frac{1}{2}=1-\omega . \end{array}$$
(4) Let *τ* = *ω*_1_ = *ω*_2_ = *ω* = 1/2. We obtain the backward EMA iterative matrix ${\tilde{S}}_{\omega ,\omega ,\omega }={\tilde{S}}_{1/2,1/2,1/2}$, and
  (52) $$\begin{array}{c} 1>\rho \left({\tilde{S}}_{\omega ,\omega ,\omega }\right)=\rho \left({\tilde{S}}_{1/2,1/2,1/2}\right)=\frac{766}{779}>\frac{1}{4}={\left(1-\omega \right)}^{2}. \end{array}$$

**Example 4**

Let the coefficient matrix *A* and the vector *b* of (1) be

(53) $$\begin{array}{c} A=\left[\begin{array}{ccc} 1 & -2 & -2\\ -1 & 1 & -1\\ -2 & -2 & 1 \end{array}\right],\;\;b=\left[\begin{array}{c} 2\\ 1\\ 2 \end{array}\right]. \end{array}$$

The Jacobi iterative matrix is

(54) $$\begin{array}{c} B=\left[\begin{array}{ccc} 0 & 2 & 2\\ 1 & 0 & 1\\ 2 & 2 & 0 \end{array}\right]. \end{array}$$

By caculation, we obtain *ρ*(*B*) = 987/305 > 1.

(1) Let *τ* = *α* = 1/2, *ω*_1_ = *ω*_2_ = *ω* = 1/4. We obtain the backward PSD iterative matrix ${\tilde{S}}_{\alpha ,\omega ,\omega }={\tilde{S}}_{1/2,1/4,1/4}$, and
  (55) $$\begin{array}{c} \rho \left({\tilde{S}}_{\alpha ,\omega ,\omega }\right)=\rho \left({\tilde{S}}_{1/2,1/4,1/4}\right)=\frac{3663}{1052}>1. \end{array}$$
(2) Let *τ* = 1, *ω*_1_ = *ω*_2_ = *ω* = 1/2. We obtain the backward PJ iterative matrix ${\tilde{S}}_{1,\omega ,\omega }={\tilde{S}}_{1,1/2,1/2}$, and
  (56) $$\begin{array}{c} \rho \left({\tilde{S}}_{1,\omega ,\omega }\right)=\rho \left({\tilde{S}}_{1,1/2,1/2}\right)=\frac{2543}{247}>1. \end{array}$$
(3) Let *τ* = *ω* = 1/2, *ω*_1_ = *ω*_2_ = 0. We obtain the backward JOR iterative matrix ${\tilde{S}}_{\omega ,0,0}={\tilde{S}}_{1/2,0,0}$, and
  (57) $$\begin{array}{c} \rho \left({\tilde{S}}_{\omega ,0,0}\right)=\rho \left({\tilde{S}}_{1/2,0,0}\right)=\frac{646}{305}>1. \end{array}$$
(4) Let *τ* = *ω*_1_ = *ω*_2_ = *ω* = 1/2. We obtain the backward EMA iterative matrix ${\tilde{S}}_{\omega ,\omega ,\omega }={\tilde{S}}_{1/2,1/2,1/2}$, and
  (58) $$\begin{array}{c} \rho \left({\tilde{S}}_{\omega ,\omega ,\omega }\right)=\rho \left({\tilde{S}}_{1/2,1/2,1/2}\right)=\frac{1530}{271}>1. \end{array}$$

It shows that the backward MPSD iteration is invalid for this example.

**Example 5**

Let the coefficient matrix *A* of (1) be

(59) $$\begin{array}{c} A=\left[\begin{array}{cccccccccc} 10 & -2 & -2 & -2 & -2 & -2 & -2 & -2 & -2 & -2\\ -1 & 10 & -2 & -2 & -2 & -2 & -2 & -2 & -2 & -2\\ -1 & -1 & 11 & -2 & -2 & -2 & -2 & -2 & -2 & -2\\ -1 & -1 & -1 & 12 & -2 & -2 & -2 & -2 & -2 & -2\\ -1 & -1 & -1 & -1 & 13 & -2 & -2 & -2 & -2 & -2\\ -1 & -1 & -1 & -1 & -1 & 14 & -2 & -2 & -2 & -2\\ -1 & -1 & -1 & -1 & -1 & -1 & 15 & -2 & -2 & -2\\ -1 & -1 & -1 & -1 & -1 & -1 & -1 & 16 & -2 & -2\\ -1 & -1 & -1 & -1 & -1 & -1 & -1 & -1 & 17 & -2\\ -1 & -1 & -1 & -1 & -1 & -1 & -1 & -1 & -1 & 18 \end{array}\right]. \end{array}$$

We can see the analogous matrix *A* in [29]. By caculation, we obtain *ρ*(*B*) = 811/822 < 1.

Let *τ* = 1, *ω*_1_ = 1, *ω*_2_ = 0. We obtain the backward Gauss-Seidel iterative matrix ${\tilde{S}}_{1,\omega_{1},\omega_{2}}={\tilde{S}}_{1,1,0}$, and

(60) $$\begin{array}{c} \rho \left({\tilde{S}}_{1,\omega_{1},\omega_{2}}\right)=\rho \left({\tilde{S}}_{1,1,0}\right)=\frac{766}{789}. \end{array}$$

The Gauss-Seidel iterative matrix $S_{1,\omega_{1},\omega_{2}}={\tilde{S}}_{1,1,0}={\left(I-L\right)}^{-1}U$, and

(61) $$\begin{array}{c} \rho \left(S_{1,\omega_{1},\omega_{2}}\right)=\rho \left(S_{1,1,0}\right)=\frac{721}{739}. \end{array}$$

Thus,

(62) $$\begin{array}{c} \rho \left({\tilde{S}}_{1,1,0}\right)<\rho \left(S_{1,1,0}\right). \end{array}$$

**Figure 6:**
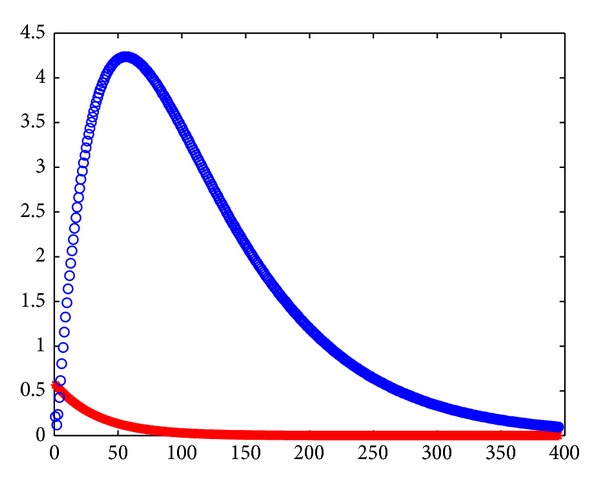
The errors of backward Guass-Seidel and Guass-Seidel iteration.

From Figure 6, the red stars denote the error of backward Guass-Seidel iteration and the blue circles denote that of Guass-Seidel. So, the backward iterative methods are better than the original methods under the assumption that the upper triangular part dominates the lower triangular part.

## 5. Conclusions

The Jacobi iteration is the basic iteration for linear systems and easier to the analysis of the convergence than other iterations. In the paper, we proposed the backward MPSD iteration and obtained the convergence result between backward MPSD iteration (including iterations such as backward JOR, backward G-S, backward EMA, and backward PSD) and Jacobi iteration. We pointed out that the backward MPSD iteration and the Jacobi iteration are either both convergent or both divergent under the assumptions in Theorem 7. So, we can ascertain the convergence or divergence of backward MPSD iteration by Jacobi iteration. In some case, the backward iteration is better than the original one.
